# Case of Abdominal Tumor, with Autopsy

**Published:** 1886-01-20

**Authors:** W. P. Nicolson


### CASE OF ABDOMINAL TUMOR, WITH AUTOPSY.

### PRESENTED TO THE ATLANTA SOCIETY OF MEDICINE BY DR. W. P. NICOLSON.

**Reported for the Southern Medical Record.**

Dr. Nicolson reported a case of a little girl, white, aged twelve years, with the following history. Nearly two years ago, while running about the room, she fell and received a blow in the right side. There was much pain, followed by fever, which kept her in bed for about two weeks. Since that time her general health has been unimpaired, but a short time after the attack she felt a "cake" above and to the right of the umbilicus. This continued to increase for about sixteen months, when she came under the care of Dr. McElroy, of South Carolina, who states that he found a distinct and large tumor occupying the space between the umbilicus and the hepatic region. This had increased until the entire abdominal cavity was filled, giving all the appearances of an ovarian cyst of large size. There was fluctuation at every point, and at no place could any resonance on percussion be elicited. The diagnosis lay between cyst of the ovary, liver, kidney or pancreas. After careful consideration the cyst had been diagnosed as pancreatic. The literature of pancreatic cysts, as reported in this country so far, had only shown two cases in which operations had been performed; one by Dr. Bozeman, who had operated under the impression that the cyst was ovarian, and the other by Dr. Senn, of Chicago. As for the reasons for pronouncing the cyst pancreatic, Dr. Nicolson stated that the idea had first been suggested by Prof. Gaston, who saw the case with him. The high origin of the tumor excluded the ovarian tumor; the absence of hooklets under the microscope showed the tumor not a echinococcus cyst, and the absence of any renal trouble had rendered a cyst of the kindey improbable. The fluid withdrawn through a hypodermic needle showed, under the microscope, blood corpuscles, cholesterine plates and cells resembling the ovarian cells. There was an emulsion formed by mixture of the fluid with oil, which favored the idea of its coming from the pancreas.

With a view to locate the stomach, a powder of bicarbonate of soda was dissolved in water and given by the mouth, followed by one of tartaric acid, which, by their reaction, developed carbonic acid gas in the stomach, and it became resonant upon percussion, indicating that it was crowded over into the right hypochondriac region, and was, consequently, pressed upon by the tumor in the epigastric region.

On December 3, 1885, G—— P——, the patient, underwent a final examination at the Ivy Street Hospital, with a view to determine upon the operation. The most salient point of this tumor being about two and a half inches below the umbilicus, the opening would have been made there, but the propriety of making an incision there was set aside by the consideration of greater density above the umbilicus, in the texture of the cyst wall, which would give more security in its attachment to the parietes; and, moreover, the sac was thought to spring from the pancreas, so that the tension after retraction would, in this case, be less from connection above than below the umbilicus.

After thorough cleansing of the surface with water and soap, a solution of bichloride of mercury was poured over the skin, and the patient being under the anæsthetic influence of sulphuric ether, Dr. W. P. Nicolson proceeded to operate by making an incision of three inches in the median line between the ensiform cartilage and the umbilicus, dividing the peritoneum upon the grooved directory, when the tense wall of the cyst came into view. A sound was passed down between the sac and the abdominal wall, which moved freely, excepting at one point in the left iliac region, where adhesion was supposed to exist, between the adjacent serous surfaces. The peritoneum was secured by continuous catgut suture to the skin around the entire abdominal opening. The tissue of the cyst was then secured with the vulsellum at two points an inch and a half distant, and a trocar was introduced, when the fluid of a light straw color flowed out until the distension was relieved; after which an incision gave a free outlet to the remaining accumulation of a consistence corresponding to that of molasses, with some cheesy flakes, upon one of which some capillary threads were discovered and attributed to shreds of woolen texture on the operating table. Dr. Bizzell, who administered the ether, remarked that the discharge resembled very much that which had been seen in an operation for ovarian cyst, but it corresponded to that described from pancreatic cysts, and hence was not regarded as pathognomonic. The edges of the incision were held out with forceps, and a sponge was kept pressed upon the lower margin of the abdominal opening, as the patient was inclined towards the left side,so as to effectually prevent the discharge from entering the peritoneal cavity. Dr. Nicolson and Dr. Gaston each examined the surface of the solid body which lay immediately within the incision of the cyst wall, by the finger, and found an irregular, nodulated mass, strengthening the previous impression of an enlarged indurated pancreatic gland, while the appearance was reddish-brown, indicating a state of hyperæmia of the glandular structure. Dr. Gaston passed his hand above and below the projecting solid tumor, occupying the epigastric region, and was satisfied, by pressure in different directions, of its mobility, while no prominence could be felt in the hypogastric region by palpation. These modes of examination and exploration seemed to warrant the carrying out of the measure originally designed, and Dr. Nicolson proceeded to stitch the margin of the incision in the sac to the edges of the abdominal wall with catgut suture, and to close the remaining opening above with strong iron-dyed silk stitches of interrupted suture. A large rubber drainage tube of half-inch diameter, having its wall fenestrated, eight inches in length, was passed into the cavity. This was manipulated so as to cause it to take a curve at the lower extremity and lie in contact with the inner surface of the cyst wall, at the most dependent part below. A strong silk ligature was passed by a needle through the projecting end and tied to a strip of rubber adhesive plaster, which was attached over the lower portion of the sternum. Iodoform gauze was now placed in contact with the wound, and over this a layer of bichloride of mercury gauze with a roller of flannel around the body to complete the dressing.

The patient rallied well and promptly, without the customary nausea and vomiting, and passed the night comfortably. Very light food was given on the following day, and on the second day she took nourishment of chicken soup with rice in moderate quantity. The temperature and pulse varied little from the normal standard during three or four days succeeding the operation. There was a free discharge around and through the drainage tube, which necessitated daily changes of all the dressings, and the applications were renewed as at first. Some flakes of cheesy matter were dislodged from the lumen of the drainage tube on the second and third days, but on the fourth the discharge no longer assumed the original characteristics, and only a clear mucous or serous fluid passed from the tube ; so that it was thought proper to partially witddraw the tube, but, upon making the attempt, it was found to be agglutinated to the orifice in such a way that moderate traction did not release it, and the tube was left in situ for another day. The discharge was much diminished during the following twenty-four hours, and Dr. Nicolson, with the concurrence of Dr. Gaston, used such force as was requisite to partially withdraw the tube on the fifth day, and this was accomplished without discomfort to the patient.

Upon visiting the patient early on the morning of December 8, there was a marked alteration in the symptoms—the pulse being 130 to the minute, and the temperature 103° Fah. Dr. Nicolson finding subsequently that the temperature rose to 104°, and the pulse 140 per minute, gave antipyrin with the result of lowering the temperature to 102° ; yet the pulse continued frequent. Increased discharge from wound.

December 9, the bowels were moved twice spontaneously, with pulse 130 and temperature 102°. Took tonic, chicken soup, and a 5-grain dose of quinine.

December 10, there were evident indications of suppuration from the cyst cavity, with temperature of 101°, and pulse 125 to the minute, accompanied by anorexia. Tonic continued during the day, and at night commenced the use of salycilate of soda, ʒi ; iodide of potash, ʒss ; extract jaborandi, fl. ʒi; simple syrup, fl. ʒvii; in teaspoonful doses every three hours; but this was attended with retching and vomiting, so that it was discontinued after the second dose. Tube was changed for smaller one.

December 11. The discharge abundant, of a dirty purulent nature, around and through the tube, which had been retracted an inch or more each day ; bowels moved spontaneously in the morning ; temperature 101½°, and pulse 125 to the minute. The catgut stiches had become disintegrated, but the margin of incision in the sac appeared to be adherent to the edges of external opening. Some of the stitches of interrupted suture in the parietal wall above this opening having cut out, others were removed, and while the peritoneal membrane was united the cutaneous structure, which previously seemed adherent, now gaped open and was closed with adhesive plaster. She took wine and beef tea during the day, in small quantities, but was thirsty and craved lemonade, and had no appetite.

December 12. There was considerable increase of the vitiated purulent discharge from the wound, and the margin presented a sloughy tendency, while the edges of the abdominal wall showed some signs of disintegration, and gaped throughout the whole extent of the incision when the adhesive plaster was removed. The smaller sized rubber tube lay loosely in the wound, and there was a flow around it as well as through it. It was drawn out and shortened about two inches, not being attached with a ligature to the adhesive plaster on the thorax as the longer tube was at the outset. The pulse was more feeble, and counted 130 to the minute, while the temperature was 102°. Was prescribed tincture of iron and quinine, with gentian, but the stomach rejected the medicine, and the patient had no inclination to take nourishment. A little wine with ice and some beef tea were the only articles retained.

December 13. The patient was found propped in a sitting posture on the bed, without any authority for such proceeding from her medical attendant, and gave evidence of great prostration of her vital powers. Her pulse was 140 to the minute, and the temperature 102°. Took Wyeth's wine, beef, and quinine, in small quantities during the day, but eventually everything was rejected by the stomach, and enemata of Reid & Carnrick's beef peptonoid were resorted to as nourishment.

It was found, upon removing the dressing, that the rubber tube had receded until the outer end of it was on a level with the cutaneous opening, and requiring to be seized with a forceps to be withdrawn, when it was attached by ligature to the plaster on the chest, and thus demonstrating the importance, under all circumstances, of securing the drainage tube externally.

A dark, grumous, degenerated sanious fluid was discharged from the wound and the tube; complained of pain in the manipulations when dressing the abdominal opening, but there was no marked distension. The adhesive plaster was continued to keep the edges of wound approximated, with other dressings, as before.

December 14. The patient sank at 6 o'clock a. m., at the expiration of ten days after the laparotomy.

The autopsy, at 3 o'clock p. m. of the same day, revealed that the lining membrane of the sac was injected and congested, with a tendency to degeneration and disintegration of the tissues composing it, while there were no signs of general peritonitis or evidence of any escape of fluid into the abdominal cavity. Upon the large indurated tumor being raised from its position upon the posterior boundary of the abdomen, it was observed that all the viscera were crowded out from behind it, to the sides as well as above and below it, and that adhesion existed at its lower part to the parietes, to the intestines, to the bladder, and, finally, that it sprang from the left ovary, the pedicle being much elongated and drawing upon the womb, which was so attenuated as to be recognized with difficulty. It was a true ovarian tumor, and the cyst proved to be developed from its surface. The error in diagnosis, owing to an incorrect history of the early formation of the protuberance, given by the former medical attendants, was, perhaps, more excusable from the flattening of the lower anterior portion of the indurated mass, and its protuberance in the epigastric region, where alone any solid projection was perceptible prior to draining off the fluid from the cyst.

The indurated tumor is of the multilocular formation, with fluids of various consistence and color in the different cavities, while portions of the mass are of a cartilaginous firmness, with here and there osseous formations, and some teeth and hair in various parts. It filled up the entire posterior aspect of the abdominal cavity, and weighed about ten pounds, with the characteristics of the dermoid degenerations, and the cyst developed on its anterior and lower portions. It is very questionable whether it could have been removed without a fatal result had the true character been discovered at the time of the operation.

